# Long non-coding RNAs in bone metastasis: progresses and perspectives as potential diagnostic and prognostic biomarkers

**DOI:** 10.3389/fendo.2023.1156494

**Published:** 2023-04-17

**Authors:** Paola Maroni, Marta Gomarasca, Giovanni Lombardi

**Affiliations:** ^1^ Laboratory of Experimental Biochemistry and Molecular Biology, IRCCS Istituto Ortopedico Galeazzi, Milano, Italy; ^2^ Department of Athletics, Strength and Conditioning, Poznań University of Physical Education, Poznań, Poland

**Keywords:** lncRNA, bone metastasis, prostate cancer, breast cancer, lung cancer, biomarkers

## Abstract

In a precision medicine perspective, among the biomarkers potentially useful for early diagnosis of cancers, as well as to define their prognosis and eventually to identify novel and more effective therapeutic targets, there are the long non-coding RNAs (lncRNAs). The term lncRNA identifies a class of non-coding RNA molecules involved in the regulation of gene expression that intervene at the transcriptional, post-transcriptional, and epigenetic level. Metastasis is a natural evolution of some malignant tumours, frequently encountered in patients with advanced cancers. Onset and development of metastasis represents a detrimental event that worsen the patient’s prognosis by profoundly influencing the quality of life and is responsible for the ominous progression of the disease. Due to the peculiar environment and the biomechanical properties, bone is a preferential site for the secondary growth of breast, prostate and lung cancers. Unfortunately, only palliative and pain therapies are currently available for patients with bone metastases, while no effective and definitive treatments are available. The understanding of pathophysiological basis of bone metastasis formation and progression, as well as the improvement in the clinical management of the patient, are central but challenging topics in basic research and clinical practice. The identification of new molecular species that may have a role as early hallmarks of the metastatic process could open the door to the definition of new, and more effective, therapeutic and diagnostic approaches. Non-coding RNAs species and, particularly, lncRNAs are promising compounds in this setting, and their study may bring to the identification of relevant processes. In this review, we highlight the role of lncRNAs as emerging molecules in mediating the formation and development of bone metastases, as possible biomarkers for cancer diagnosis and prognosis, and as therapeutic targets to counteract cancer spread.

## 1 Introduction

In the past, non-coding RNAs (ncRNAs) were poorly considered both because of the lack of coding potential and because they were considered only intragenic transcripts and, therefore, “transcriptional noise”, or “junk” RNA. Currently, their versatile roles are recognized in numberless cellular processes, from physiological development to pathological processes, thus becoming an area of interest for researchers. ncRNAs include micro RNAs (miRNAs), long non-coding RNAs (lncRNAs), circular RNAs (circRNAs), and small interference RNAs (siRNAs).

In particular, ncRNAs can be categorized according to their size into small ncRNAs, that include transcripts from 19-25 nucleotides to <200 nt in length, such as miRNAs and siRNAs, but also P-element-induced wimpy testis (PIWI)-associated RNA (piRNA), small nucleolar RNA (snoRNA), and long ncRNAs, comprising transcripts longer than 200 nucleotides (1).

LncRNAs family grouped heterogeneous members that participate in fundamental cellular processes, such as high-order chromosomal dynamics, telomere biology, subcellular structural organization and, as recently emerged, involvement in the regulation of the expression of neighboring protein-coding genes (2). Deregulation or aberrant expression of lncRNAs affects these key processes and, in turn, impacts on high-level cellular responses such as cell fate decision, immune response, cancer cell proliferation and metastasis (3).

In general, lncRNAs are considered master transcriptional regulators (4, 5). Different subcellular localizations of lncRNAs dictate their mechanism of action and the specific function. In the nucleus, once transcribed, lncRNAs can stay anchored to the chromatin at the site of transcription or accumulate in a distant location. Accordingly, they can act as gene expression guides either in cis, to control local gene expression, or in trans, to control distant gene expression (6, 7). In the nucleus they regulate chromosome structure (8), participates in chromatin remodeling (9), adjust binding domains for the transcription machinery, interact with repressor complexes or block the transcription starting site, thus either activating or repressing gene expression. When lncRNAs are exported into the cytoplasm, they regulate mRNA stability (10), translation (11), and interfere with post-translational modifications (12), as well as regulates miRNA expression and function (13). Therefore, lncRNAs have important functions in regulating gene expression at different levels: epigenetic, transcriptional, and post-transcriptional (14).

In recent decades, the crucial role of lncRNAs has been related to their ability to influence a variety of processes that accompany normal and cancerous cell biology.

The possible roles of lncRNAs in tumorigenesis have been reported in many studies. Tumor cells can secrete lncRNAs into biological fluids (intercellular fluid, blood, urine, saliva, cerebrospinal fluid, etc.) within microvesicles or exosomes, or associated in protein complexes and, being in a stable state protected from ribonuclease-mediated degradation, can move as circulating lncRNAs. The secretion of lncRNAs in the tumor microenvironment is also important to impinge on the activity of different immune and stromal cells that infiltrate the tumor microenvironment and in this way, lncRNAs participate in cancer progression. In cancer patients, aberrant expression of lncRNAs has been reported and, in this context, endogenous lncRNAs can control the expression of oncogenes associated with their suppressive and/or oncogenic functions (15).

Bone metastases represent a leading cause for the high mortality rate among patients with breast, prostate, and lung cancers. Bone metastases represent the last, and often incurable, stage of these primary tumors, mainly due to the limited therapeutic options. Treatment of bone metastasis typically involves a combination of systemic therapies (such as chemotherapy, hormone therapy, or immunotherapy) and local therapies (such as radiotherapy or surgery). Currently, the only FDA approved therapies for bone metastasis are two anti-resorptive agents the bisphosphonate (zoledronate and ibandronate) and the anti-RANKL monoclonal antibody (denosumab), and a bone-targeted radioisotope (radium 223). The anti-resorptive agents act by suppressing the osteoclasts’ function; in this way the vicious cycle between osteoclasts and cancer cells is impaired. The bone-targeted radioisotopes, radium-223, is directed to osteosclerotic lesions and is approved by FDA for the treatment of castration-resistant prostate cancer (16). Taken together, these compounds act by suppressing the osteoclast functions and are aimed to slow bone resorption to reduce skeletal related events (SREs), to improve the patient’s quality of life, relieving pain, instability, and paralysis. Metastases severely impair the quality of life and reduce the survival of patients, and remain one of the most important public health problems worldwide.

Preclinical and clinical studies are still ongoing to evaluate additional and more relevant therapeutic targets to successful treat bone metastasis.

The exact mechanisms by which lncRNAs affect tumor progression, metastasis development, and drug resistance have not been fully described and still require in-depth studies. However, over the past few decades, a great deal of precious information about the roles of lncRNAs in these processes has been produced.

This review, in particular, takes stock of the information regarding the roles of lncRNAs on the development of bone metastases, provides new insights into the complexity of bone metastases, and support the possibility to use lncRNAs as molecules with great potential as biomarkers and targets in the therapy of bone metastases.

## 2 lncRNAs classification

lncRNAs are transcribed mainly by RNA polymerase II, following they are 5′ capped, spliced, and 3′ polyadenylated, but as result they do not encode proteins because of the lacking of an open reading frame (ORF) (17). However, in some cases small ORFs exist within lncRNAs, giving rise to short functional polypeptides that may exert a variety of biological functions, even in cancer (18).

**Figure 1:**
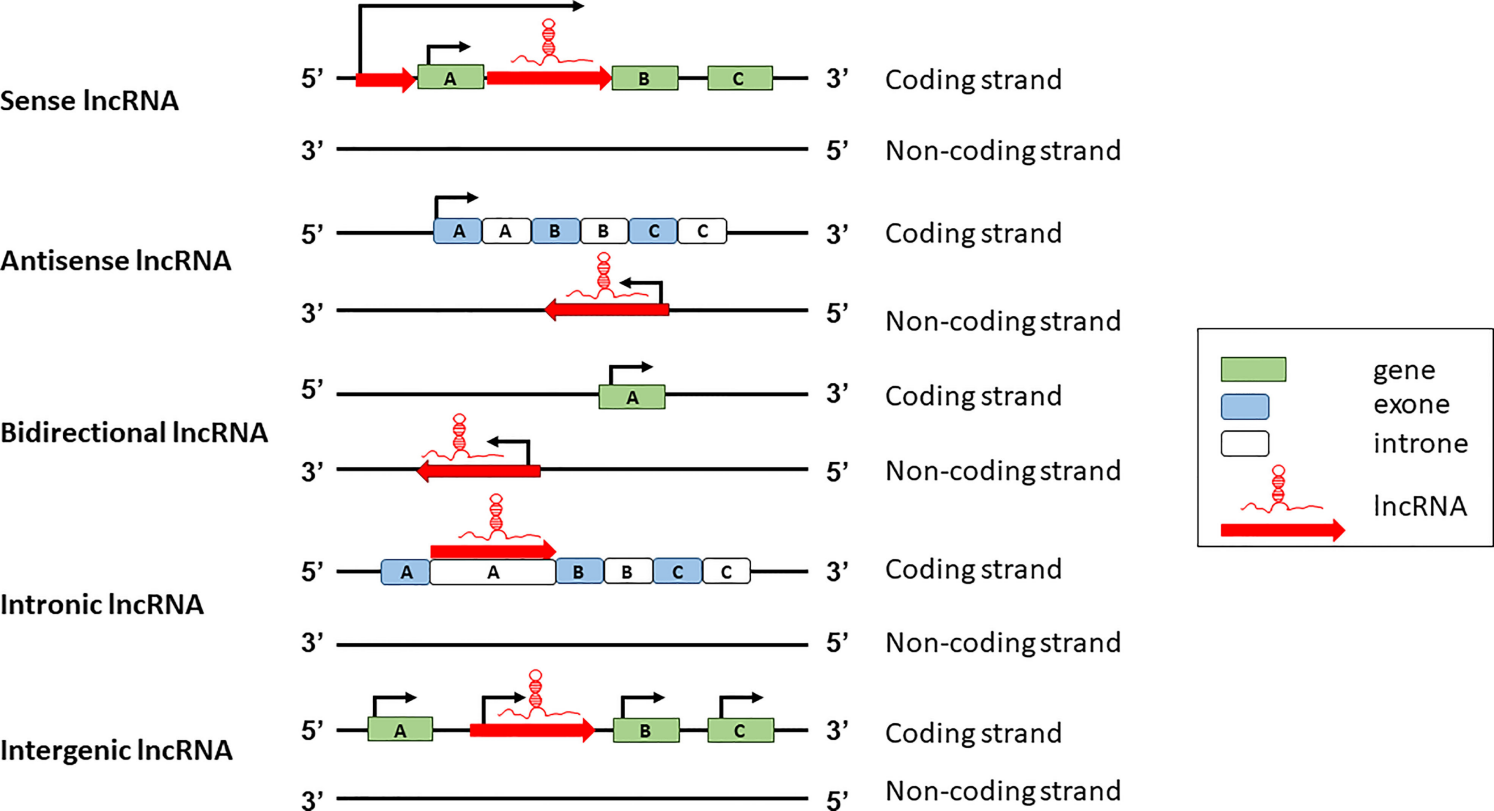
Classification of lncRNA based on genomic structure and position.

lncRNAs can be classified according to two major schemes, one based on their genomic organization and the other based on their function. Based on the genomic organization, lncRNAs can be classified in five main categories: sense, antisense, intergenic, intronic, and bidirectional (**Figure 1**). Sense lncRNAs are transcribed in the same direction as protein coding genes; antisense lncRNAs are transcribed in the antisense direction of overlapping protein-coding genes; bidirectional lncRNAs are transcribed from the promoter regions of protein-coding genes in a bidirectional manner; intronic lncRNAs have the entire sequence falling in the intronic region of protein-coding genes; intergenic lncRNAs are transcribed from the intergenic region between two protein-coding genes (19).

**Figure 2:**
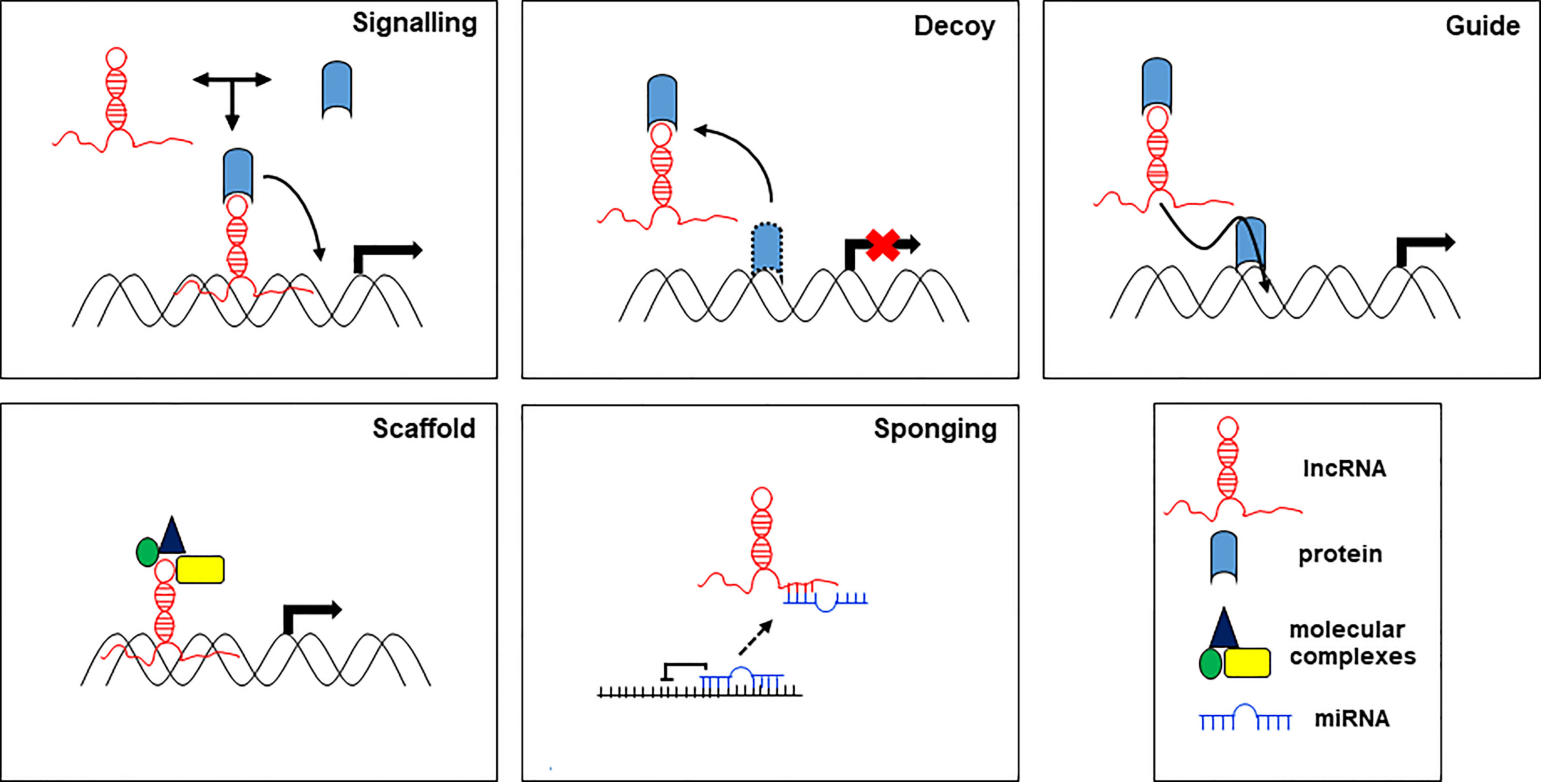
Main proposed working mechanisms for lncRNA based on their function.

On the other hand, lncRNAs can act through different molecular mechanisms, which, based on their mode of action, are grouped in 5 categories: signals, decoys, guides, scaffolds, and sponges (**Figure 2**).

lncRNA as signaling molecules integrate developmental signals by interacting with proteins, RNA, and lipids, and respond to different stimuli. lncRNAs provide a background to smooth or make more flexible the signals in response to environmental cues. lncRNAs as molecular decoys can bind and sequester transcription factors (proteins) or miRNAs (RNA) to regulate transcription and translation of their targets. As decoy, lncRNAs preclude the access of regulatory proteins to DNA. lncRNAs as guide, may act within the nucleus, where they drive chromatin modifiers and transcription factors to DNA to regulate the expression of target genes, and in the cytoplasm, where they bring together proteins and mRNAs to regulate the translation of these transcripts. As scaffolds, lncRNAs can group together multiple proteins to form ribonucleoproteins (RNP) complexes. Moreover, scaffold lncRNAs play a structural role by providing a platform for the transient assembly of multiple enzymatic complexes and other regulatory co-factors. lncRNAs function as molecular sponges for miRNAs, controlling their bioavailability and preventing their binding to the targets, thereby abolishing post-transcriptional regulation (20). In this function, lncRNAs act as competitive endogenous RNAs (ceRNAs) to regulate the abundance and the activity of other RNAs by forming the regulatory network lncRNA-miRNA-mRNA. Aberrations in the ceRNA network have been identified in human diseases including cancer. The action of lncRNAs in the regulation of target miRNAs aggregation and biological functions affects tumorigenesis and disease progression (13).

lncRNAs regulate gene transcription through five main mechanisms: (i) as chromatin regulators, lncRNAs interact and recruit chromatin-modifying enzymes (e.g., histone methylases, acetylases, and deacetylases) to the target gene locus and determine activation or repression of the local genes, (ii) as transcriptional regulators, lncRNAs interact with other RNA-binding factors (such as heterogeneous nuclear ribonucleoproteins, hnRNPs), and act as co-factors to modify the activity of transcriptional factors, (iii) as post-transcriptional regulators, lncRNAs also have enhancer or repression functions. As enhancers, they change the chromatin architecture and recruit transcriptional machinery proteins to adjacent target gene locus to promote its transcription. As decoy, lncRNAs interact with some transcription factors to limit expression of pro-apoptotic genes. (iv) As epigenetic regulators, lncRNAs solve important functions related to the epigenetic control of specific target genes, (v) as signal transducers, lncRNAs, encapsulated in extracellular vesicles, can shuttle from one site to another and, in the case of cancer, mediate the exchange of information between tumour cells as well as the crosstalk between tumour cells and stromal cells, not only in the primary site of growth but also in distant organs, thereby influencing the formation of the pre-metastatic niche.

The capacity of lncRNAs to bring regulatory molecules (e.g., mRNAs, miRNAs, DNA) in proximity of proteins (e.g., chromatin-modifying complexes, transcription factors, E3 ligases and RBPs) explains their scaffold function, which by favouring the interactions, modulates cellular activity (21, 22).

## 3 The roles of lncRNAs in cancer progression

lncRNAs are involved in every step of cancer development and progression by regulating important cancer hallmarks (23): cell proliferation (24), tumor angiogenesis (25), epithelial-mesenchymal transition (EMT) (26), distant metastasis development (27), and chemotherapy resistance (28).

lncRNAs are involved in most of the important biological processes: cell cycle, cell differentiation, development, and pluripotency (29).

lncRNAs may exert either pro- or anti-tumorigenic effects and, therefore, based on their influence on tumour onset and development they can be grouped as oncogenic and tumour suppressor transcripts (30). Between the tumour suppressor lncRNAs, there are those directly or indirectly regulated by p53 (i.e., lincRNA-p21, LINC-PINT lncRNA, LOC285194) (31–33). Other lncRNAs with tumour suppressor characteristics for certain type of cancers are: X–inactive specific transcript (XIST) lncRNA, a tumour suppressor in breast, cervical and ovarian cancers (34); GAS5 lncRNA that regulates cell proliferation, growth arrest, and apoptosis (35); CTD903 lncRNA that inhibits EMT (36); TUSC7 lncRNA that is involved in transcription and regulation of miRNAs (37); MALAT1 a tumor suppressor lncRNA downregulated in breast cancer (38). Numerous lncRNAs function as oncogenes in different types of cancer: NEAT1 has been reported function as oncogene in breast tumours, endometrial carcinoma and osteosarcoma (39–41); HOTAIR promotes tumorigenesis by inducing the triggering of EMT and the stemness acquisition (42); antisense non-coding RNA in the INK4 locus (ANRIL) induces tumor invasion and progression and participates to the epigenetic silencing of p15 and p16, two-tumor suppressor (43).

**Figure 3:**
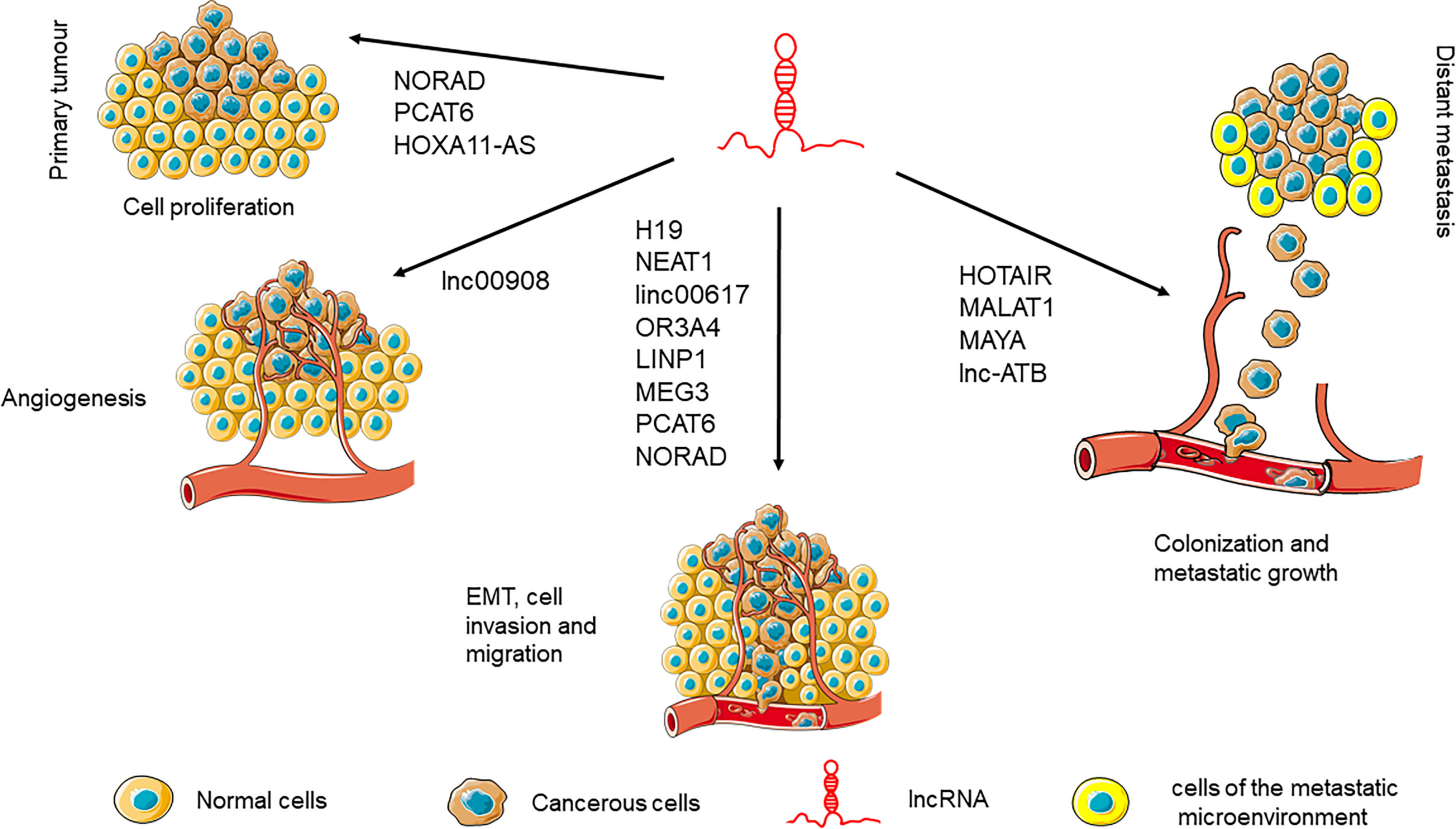
Main lncRNAs involved in cancer progression.

The role of lncRNAs during the complex process of tumorigenesis, starts from the early stages that involve translational deregulation and genomic instability, through proliferation imbalance. A schematic representation of the more relevant lncRNAs during the different phases of the metastatic process is illustrated in **Figure 3**.

### 3.1 Proliferation

The process of carcinogenesis is associated with phenotypic changes of the neoplastic cell, such as EMT and cell migration, that contribute to the definition of local hypoxic regions that promote survival and growth of the neoplastic cells, and stimulate angiogenesis. Also autophagy, a cellular process altered during the neogenesis of tumor, promotes survival of neoplastic and tumor cells under condition of stress. While in normal cells growth and survival are under the control of growth factor and hormones, in tumor cells they are promoted by constitutively activated pathways that favor proliferation and by hijacking apoptotic pathways. These altered signaling pathways result from mutations and epigenetic changes that eventually make the cells resistant and independent from these pathways. In turn, altered signaling pathways together with metabolic changes, lead to epigenetic modifications in the gene expression patterns of the cells (44).

Importantly, epigenetic changes that regulate the neoplastic formation are modulated by ncRNAs, and among them, lncRNAs. For example, it was demonstrated that the overexpression of lncRNA MEG3 led to inhibition of proliferation and metastasis in gastric cancer through the enhancement of p53 expression (45). LncRNA SPINT1-AS1 was shown to promote breast cancer cell proliferation and migration by sponging miR-let-7a/b/i-5p, thus acting as an important regulator of breast cancer progression (46). Similarly, lncRNA-CDC6 functions as ceRNA by sponging miR-251 to regulate the expression of CDC6, and thus it promotes proliferation in breast cancer cells and favors metastasis formation (47). Other lncRNAs have been demonstrated to promote proliferation, invasion and migration in different tumors by modulating the function of specific miRNAs (48, 49).

### 3.2 Epithelial-mesenchymal transition

EMT, a crucial step in metastatic process, is characterized by the phenotypic conversion of epithelial cells into mesenchymal-like cells with strong capacity to move and invade the surrounding tissues. In the field of cancer metastasis, the crosstalk between lncRNAs and EMT regulators is an important topic of discussion. It has been reported that lncRNAs act, as decoy molecules, to promote or inhibit cancer metastasis by sequestering miRNA involved in EMT (50).

A group of EMT-related transcription factors namely Snail, Slug, ZEB1, ZEB2, and Twist, activated during EMT can be modulated by lncRNAs similarly to what happens to signals originated from the tumor stroma (e.g., TGFβ, EGF, FGF, PDGF, HGF and their downstream signaling). In the late-stage tumor, TGFβ promotes cancer metastasis through the activation of EMT process and lncRNAs can regulate TGFβ signaling pathway. Most of the identified lncRNAs promote EMT, while only a minor number of them is reported to suppress EMT, but there are some lncRNAs that function as pro-EMT in certain cancers while in other they possess anti-EMT activities.

EMT is a complex bidirectional process; when cells reach the secondary growth site the reverse process, the mesenchymal-epithelial transition (MET) process occurs; therefore, it is difficult to identify the exact moment when to target either process to inhibit metastasis development (51).

### 3.3 Angiogenesis

Angiogenesis consists in the development of new blood vessels from pre-existing ones that, by providing the adequate nutritional support for the growing mass, is necessary for tumour progression and metastasis formation. lncRNAs can regulate directly or indirectly various processes involved in angiogenesis through the regulation of key molecules, known to play important roles in angiogenesis, such as vascular endothelial growth factor (VEGF) (52). In addition to the interactions with proteins, lncRNAs affect angiogenesis through the intricate crosstalk with miRNAs and mRNAs in tumour cells. lncRNAs, therefore, can adopt diverse mechanisms to stimulate tumour cells to release angiogenic factors that, in turn, induce endothelial cells to enhance the capillaries network within tumours useful for cancer development and spread (25).

### 3.4 CSCs generation

Cancer stem cells (CSCs) are highly aggressive cancer cell populations with the ability to self-renew and capable of generating differentiated tumor offspring, characteristics that are responsible for maintenance and promotion of cancer progression. EMT program can be considered as one of several components of stemness attributed to CSCs, and an essential feature of CSCs to invade, disseminate, and, eventually, metastasize. Given the close relationship between EMT and CSCs, it is not surprising that many of the lncRNAs regulating EMT are also associated with regulation of CSCs phenotype, biogenesis, and maintenance by interacting, either directly or indirectly, with a series of core pluripotency and self-renewal transcription factors including SOX2, OCT4, NANOG, KLF4, and LIN-28 (53).

Importantly, CSCs significantly correlate to cancer cell resistance to therapy and, also in this process, lncRNAs are significantly involved (54).

It has been recently demonstrated that a specific lncRNA, namely lncROPM (a regulator of phospholipid metabolism), is responsible for the maintenance of stemness and resistance to chemotherapeutics in breast cancer stem cells, through the regulation of lipid metabolism (55). Similarly, another lncRNA termed PKMYT1AR (PKMYT1 associated lncRNA) has been found involved in CSCs maintenance in NSCLC through the inhibition of ß-catenin ubiquitination, thus, stimulating tumorigenesis (56).

## 4 The roles of lncRNAs in bone metastasis

Metastatic process is characterized by a multistep cascade: it starts early with the detachment of the tumour cells from the primary site, then continues through local invasion, induction of angiogenesis, intravasation, survival into the bloodstream, extravasation, and, finally, the attainment by cancer cells of the distant target tissues where they can grow to give rise to a metastatic lesion.

Bone is the primary and most common site where secondarisms develop from breast, prostate, and lung cancers. Bone is a peculiar, highly active and dynamic tissue with high blood flow in the red marrow; it provides mechanical support and produces angiogenic and bone resorption factors useful for tumour growth. As cells spread to bone, they can proliferate to generate secondary growth or they can remain in a dormancy phase for years, as is often the case of metastatic breast cancer cells. After a prolonged time, even years, when the microenvironment conditions become permissive, the metastatic cells hosted in the bone marrow begin to proliferate again, giving rise to macrometastases.

The incidence of bone metastasis varies depending on the type of cancer and the stage of the disease: in the advanced stage bone is the most common site of metastasis for breast cancer with up to 75% of patient developing bone metastasis, in prostate cancer, bone metastasis occurs in up to 70-85% of patients and in lung cancer, bone metastasis occurs in approximately 40% of patients (57). The risk of developing bone metastasis increases with age. This is likely due to a combination of factors, including increased cancer incidence with age and age-related changes in bone structure and function that may facilitate cancer cell growth and invasion.

There is, therefore, an urgent need to identify new biomarkers for the early detection of aggressive tumours, in order to prevent the metastasis formation or to keep spread cells in the dormancy phase and, thus, to avoid the recurrence of the disease.

The role of several lncRNAs has recently been reported as primary regulators of bone metastasis formation and inducers of distant organ colonization by metastatic cells. As introduced above, lncRNAs promote invasion and metastasis by acting on different processes: by inducing EMT, by acting on cell adhesion molecules and MMPs, through the regulation of miRNA, by modulating cell cycle and apoptotic pathways (58). Below we give an overview of the main lncRNAs involved in bone metastases focusing on prostate, breast and lung cancer, the main types of cancer that metastasize to the skeleton.

### 4.1 Prostate cancer bone metastasis

Sost, an osteocyte-derived Wnt inhibitor, was found to be deregulated in osteoblasts in an *in vitro* model of osteoblasts-prostate cancer interactions, and that Sost acted as major regulator of gene expression in highly invasive PC3 cells. Among the genomic sequences regulated by Sost, lncRNA MALAT1 was found to be directly targeted. This indicates that reduced Sost expression in the tumour microenvironment may promote bone metastasis by up-regulating MALAT1 in prostate cancer (59).

The nuclear-enriched abundant transcript 1 (NEAT1) is a lncRNA overexpressed in many human cancer types (60), and its enhanced expression is associated with a worse prognosis in cancer patients.

Several studies have reported that 6-methyladenosine (m6A) participates in the post-transcriptional modifications of mRNAs and ncRNAs (e.g., miRNAs, lncRNAs, and circRNAs) always associated with tumorigenesis, cancer progression, and metastasis development, where they are crucial molecular regulators. m6A modifications can act as a structural “switch” in the modulation of RNA fate at various levels to change the conformation of lncRNAs. The m6A modifications have regulatory effects on noncoding RNAs but also ncRNAs can regulate m6A modifications in biological processes (61). In this context, NEAT1/m6A levels were positively correlated with prostate cancer progression and bone metastases onset, and negatively correlated with patient survival. NEAT1/m6A has been reported to facilitate the oncogenic function of the novel CYCLINL1/CDK19 complex in patient-derived metastatic bone xenograft (PDX) and that NEAT1/m6A plays a vital role in regulating the progression of prostate cancer. This epigenetic modification could therefore represent a novel target for prostate cancer therapy and diagnosis (61).

In a recent study, it has been demonstrated that NEAT1, transferred by PCa-derived exosomes, exerts effect on human bone marrow‐derived mesenchymal stem cells (hBMSCs) by inducing osteogenic differentiation, by altering the expression of RUNX2 through a competitive binding with miR-205-5p and the regulation of splicing factor proline- and glutamine-rich (SFPQ)/polypyrimidine tract-binding protein 2 (PTBP2) axis. These findings pave the way for the development of an efficient therapeutic strategy to counteract bone metastasis in PCa patients (62).

The lncRNA prostate cancer‐associated transcript 6 (PCAT6) is significantly upregulated in PCa tissues with bone metastasis and is correlated with poor survival in PCa patients. PCAT6 knockdown *in vitro* inhibited PCa cell migration, invasion, and proliferation, and counteracted tumour growth and bone metastasis development *in vivo* (63). The modification of PCAT6 with m6A determine the upregulation of PCAT6 in bone metastasis‐positive prostate cancer, the interaction with IGF2BP2 to stabilize IGF1R mRNA, the upregulation of IGF1R expression, and the promotion of PCa cell invasion, migration, tumour growth and bone metastasis formation (63). Similarly, the lncRNA prostate cancer‐associated transcript 7 (PCAT7) was elevated in bone metastasis‐positive PCa tissues. Indeed, PCAT7 mediated the activation of TGFβ/SMAD signaling through the up-regulation of TGFBR1 expression, *via* sponging miR‐324‐5p. In turn, SMAD3/SP1 transcriptional complex up-regulated PCAT7 transcription, determining a feedback loop that promotes prostate cancer bone metastasis (64).

Misawa and colleagues found that homeobox A11 antisense RNA (HOXA11-AS), a highly expressed lncRNA in cell lines derived from prostate cancer bone metastases, promoted invasion and proliferation in PC3 cells. The authors identified lncRNA transcription factor homeobox B13 (HOXB13) as an upstream regulator of HOXA11-AS and demonstrated that HOXB13/HOXA11-AS axis regulates the production of CCL2/CCR2 cytokines and integrin signaling in bone metastatic prostate cancer cells, while HOXA11-AS secreted from PC3 cells modulates CCL2/CCR2 cytokine signaling in osteoblasts present in the bone marrow. The authors suggested that the lncRNA HOXA11-AS, together with its transcription factor HOXB13, regulates the bone tropism of prostate cancer cells through specific downstream cytokine and integrin signals (65).

A functional significance of the lncRNA KCNQ1OT1/miR-211-5p/CHI3L1 axis has been demonstrated in two PCa cell lines, DU145 and LNCaP: lncRNA KCNQ1OT1 sponged miR-211-5p with subsequent upregulation of chitinase-3-like-1 (CHI3L1), and the promotion of malignant progression of PCa. These findings suggested a role for lncRNA KCNQ1OT1 as a ceRNA in the promotion of proliferation, invasion, and bone metastasis of PCa cells (66).

lncRNA activated by DNA damage (NORAD), highly expressed in prostate cancer cell lines, where it promotes proliferation and invasion, has been reported to promote also bone metastasis. In prostate cancer cells, the interaction between NORAD and miR-541-3p promotes bone metastasis formation through the induction of pyruvate kinase isozymes M2 (PKM2) expression. This induction results in the enhanced secretion and internalization, by PCa cells, of extracellular vesicles (EVs), the key mediators of the crosstalk between tumour cells and distant metastatic organs. However, the mechanism by which EVs-PKM2 affects BMSCs to promote bone metastases has yet to be determined (67).

lncRNA small nucleolar RNA host gene 3 (SNHG3) has been implicated in the initiation and progression of multiple human cancers. By targeting several miRNAs, SNHG3 stimulates tumour development: it has been reported that, in prostate cancer cells metastasizing to the bone, SNHG3 might serve as a miR-214-3p sponge to increase TGFBR1 expression and, by activating TGF-β signaling pathway, stimulates tumour growth and bone metastasis development. Therefore, in prostate cancer cells, SNHG3 can be considered a prognostic biomarker and a promising target against bone metastases. However, the mechanism by which the lncRNA SNHG3 is upregulated remains currently unknown (68).

The oncofoetal lncRNA H19, one of the first discovered lncRNAs, plays a key role during tumorigenesis starting from the early stages involving translational deregulation and genomic instability, to proliferation imbalance and stress management, to metastasis (69). H19 can exert either a promoting role in oncogenesis (70–72) or a tumor suppressor role (73, 74), depending on the cell type and the established tumour microenvironment. In metastatic PCa cells, H19 is downregulated compared to non-metastatic PCa cells and acts as a suppressor of prostate cancer metastasis *via* H19/miR-675 axis through the downregulation of TGFB1 (75). However, the pathophysiological role of H19 in this type of cancer is not clearly elucidated. Nanni and colleagues demonstrated that oestrogen and hypoxia signaling synergistically promote a specific gene transcription program, which allows the acquisition of an aggressive phenotype in prostate cancer cells (76). The same researchers showed that H19 was induced by oestrogen and hypoxia if applied as separated stimuli, while, on the contrary, the combined treatment (hypoxia plus oestrogen) counteracted H19 expression. H19 acts as a transcriptional repressor of cell adhesion molecules, in fact during H19-silencing or combined treatment both β3 and β4 integrins and E-cadherin were up-regulated. Additionally, combined treatment increased both cell motility and invasion of PCa cells indicating that oestrogen and hypoxia, *via* H19, transcriptionally regulated cell adhesion molecules, redirecting metastatic dissemination from EMT to a β integrin-mediated invasion. This mechanism potentially identifies an H19/integrin pathway to be considered as a new target for molecular therapy design (77).

Prensner and colleagues identified lncRNA SChLAP1 as a potential biomarker useful to predict metastatic progression in prostate cancer patients treated with radical prostatectomy. These authors showed a non-invasive method to detect SChLAP1 in urine samples suggesting that SChLAP1 could represent a promising biomarker to discern aggressive prostate cancer (78).

Recent study elucidate the role of lncNAP1L6 on PCa progression. lncNAP1L6 is highly expressed in PCa cells and promotes cell migration, invasion and EMT. lncNAP1L6 upregulates the expression of METTL14/METTL3 and METTL14/METTL3 complex induces m6A methylation of NAP1L2 (Nucleosome Assembly Protein 1 Like 1), a nearby gene of NAP1L6. In addition, lncNAP1L6 through the HNRNPC protein, stabilizes the NAP1L2 mRNA and stimulates the interaction of NAP1L2 with YY1 which activates the MMP pathway facilitating the malignant progression of PCa. This study reveals that lncNAP1L6 might be used as potential therapeutic target in PCa (79). Finally, it has been reported that HOX transcript antisense RNA (HOTAIR) is up-regulated in PCa bone metastatic tissues compared with matched primary PCa tissues, by an ISH assay, and that serum level of HOTAIR positively associated with that of bone metabolic markers (elevated in patients with PCa bone metastasis) (80). These authors proposed HOTAIR as potential biomarker and therapeutic target for PCa bone metastasis. The mechanism of action of HOTAIR in PCa cells is related to its capacity to sponge miR-520b and to up-regulate of FGFR1 expression (**Table 1**).

**Table 1:** Lists of the lncRNAs involved in the bone metastatic process in prostate, breast, and lung cancers.

Cancer type	lncRNAs	Role	Targets or modulated molecules	Function	References
**Prostate cancer**	MALAT1	Signal	Unknown	Downregulated by sclerostin	(59)
	NEAT1	Signal	CYCLINL1/CDK19	Stimulates oncogenic functions	(61)
	NEAT1	Decoy	miR-205-5p	Alters the expression of RUNX2	(62)
	PCAT6	Signal	IGF1R	Stimulates cell migration, invasion, and proliferation	(63)
	PCAT7	Decoy	miR-324-5p	Activates TGFβ/SMAD signaling	(64)
	HOXA11-AS	Signal	CCL2/CCR2 and integrin	Regulates the bone tropism	(65)
	KCNQ10T1	Molecular sponge	miR-211-5p	Up-regulates CHI3L1 and promotes malignant progression	(66)
	NORAD	Molecular sponge	miR-541-3p	Induces PKM2	(67)
	SNHG3	Molecular sponge	miR-214-3p	Activates TGFβ signaling pathway	(68)
	H19	Molecular sponge	miR-675	Down-regulates TGFβ1	(75)
	SChLAP1	Signal	Unknown	Discerns aggressive prostate cancer	(78)
	NAP1L6	Signal	METTL14/METTL3	Unknown	(79)
	HOTAIR	Molecular sponge	miR-520b	Up-regulates FGFR1 expression	(80)
**Breast cancer**	MALAT1	Decoy	miR-448	Unknown	(81)
	HOTAIR	Molecular sponge	miR-20a-5p	Induces BC development and tumorigenesis	(82)
	SNHG3	Molecular sponge	miR-1273g-3p/BMP3	Induces proliferation, migration, and BMP3 expression	(83)
	DLX6-AS1	Molecular sponge	miR-505-3p	Targets RUNX2	(84)
	MAYA	Signal	CTGF	Promotes osteolytic bone metastasis	(85)
	ATB	Decoy	miR-200c	Up-regulates ZEB1 and ZNF217, and induces EMT	(86)
	MIR4435-2HG	Signal	Unknown	Drives EMT	(87)
	NEAT1	Signal	Unknown	Induces EMT, decreases E-cadherin expression	(88)
	linc00617	Signal	Unknown	Induces EMT, decreases E-cadherin expression	(89)
	OR3A4	Signal	Unknown	Induces EMT, decreases E-cadherin expression	(90)
	LINP1	Signal	Unknown	Induces EMT, decreases E-cadherin expression	(91)
	FGF14-AS2	Signal	eIF4E/eIF4G complex	Reduces the transcription of RANKL	(92)
**Lung cancer**	PXN-AS1	Signal	PXN	Enhances cell viability, proliferation and migration; inhibits apoptosis	(93)
	MALAT1	Signal	Unknown	Induces proliferation, migration and invasion	(94)
	SOX2OT	Signal	miRNA-194-5p/RAC1	Regulates osteoclasts differentiation	(95)
	HOTAIR	Signal	TGFβ/PTHrP/RANKL	Regulates osteoclasts differentiation and bone resorption	(96)

CDK19, cyclin-dependent kinase 19; IGF1R, insulin-like growth factor 1 receptor; TGFβ, transforming growth factor β; CCL2, CC motif chemokine ligand 2; CCR2 CC motif chemokine receptor 2; CHI3L1, chitinase-3-like 1; FGFR1, fibroblast growth factor receptor 1; BC, breast cancer; BMP3, bone morphogenetic protein 3; CTGF, connective tissue growth factor; ZEB1, zinc finger E-box binding homeobox 1; ZNF217, zinc-finger protein 217; EMT, epithelial-mesenchymal transition; eIF4E/eIF4G, eukaryotic Initiation Factor 4E/eukaryotic Initiation Factor 4G; PXN, paxillin; RAC1, Ras-related C3 botulinum toxin substrate 1; PTHrP, parathyroid hormone-related peptide; RANKL, receptor activator of nuclear factor κB ligand.

### 4.2 Breast cancer bone metastasis

lncRNA MALAT1 levels are high in luminal subtype while relatively low in triple negative breast cancer (TNBC) subtypes. However, although MALAT1 is less expressed in TNBC, in these kind of cancers it plays crucial role in regulating the expression of key genes involved in tumour progression and metastasis. These results support the potential of monitoring MALAT1 levels as a prognostic predictor of tumour recurrence and metastasis to better stratify TNBC patients with regard to their risk of disease progression (81).

In breast cancer tissue and cells, the lncRNA HOTAIR was upregulated, and its knockdown inhibited cell propagation, metastasis and facilitated cell apoptosis. The increased expression lncRNA HOTAIR in primary tumours is a powerful predictor for metastasis onset and clinical outcome (9). lncRNA HOTAIR acts as a molecular sponge of miR‐20a‐5p and, as ceRNA, it affects the activity and regulates the miR‐20a‐5p target genes, like HMGA2, thus contributing to breast cancer (BC) development and tumorigenesis (82). Sorensen and colleagues evaluated the potential role of lncRNAs in breast cancer prognosis and identified HOTAIR as an independent prognostic marker of metastases. They provide additional useful information for the identification of patients, carriers of oestrogen receptor-positive primary breast cancer, eligible for adjuvant therapy and for the reduction of overtreatment of patients that who do not need it (97).

lncRNA SNHG3 is overexpressed in both BC tissue and in BC metastatic cells and this might be responsible for the occurrence of osteolytic metastasis. Knockdown of SNHG3 prevented BC cells proliferation and migration, and inhibited the bone morphogenetic protein 3 (BMP3) expression in bone-derived mesenchymal stem cells through the up-regulation of miR-1273g-3p. These results implied the potential of SNHG3/miR-1273g-3p/BMP3 axis as novel target to treat BC bone metastases (83).

The up-regulation of DLX6-AS1 in both breast cancer tissues and cell lines is important for breast cancer progression. In particular, it has been reported that DLX6-AS1 can act as a ceRNA to promote carcinogenesis through the reciprocal negative modulation of miR-505-3p in BC cells by directly targeting RUNX2 (84).

Liu et al. developed a complete protein interaction map by constructing a ceRNA network and gene expression profile in patients with breast cancer and demonstrated that DLX6-AS1 may be a key contributor to bone metastasis in patients with advanced breast cancer. These results highlight the importance of DLX6-AS1 in the expression and regulation of miRNAs (98).

A crosstalk between receptor tyrosine kinases-like orphan receptors 1 (ROR1)-HER3 and the Hippo-YAP pathways has been reported in breast cancer and is related to the development of bone metastasis. In particular, this crosstalk is mediated by lncRNAs under cancer-specific context. MST1/2-Antagonizing for YAP Activation (MAYA) promotes osteolytic bone metastasis in breast cancer. Knockdown of MAYA in breast cancer cells with bone tropism (BoM-1833 cells) counteracts connective growth factor (CTGF) secretion and cancer cell-induced osteoclast differentiation and, consequently, limits the potential for osteolytic bone metastasis. In a murine xenograft model of bone metastasis (with both breast cancer or lung cancer cells) MAYA knockdown reduced bone metastasis burden suggesting that targeting MAYA is a possible strategy to impair bone metastasis development (85).

lncRNA activated by TGF-β (lnc-ATB) acts as a key regulator of TGF-β signaling pathway and promotes Trastuzumab resistance and invasion-metastasis cascade in breast cancer by competitively biding miR-200c, up-regulating ZEB1 and ZNF-217, and then inducing EMT (86).

lncRNA MIR4435−2HG expression in breast cancer cells promotes metastasis by driving EMT. It has been reported that lncRNA MIR4435−2HG knockdown decreases the expression of mesenchymal markers (N−cadherin, vimentin, and ZEB1), whereas it augments expression of epithelial marker as E−cadherin (87). Moreover, knockdown of LncRNA MIR4435−2HG results in the inactivation of the Wnt/β-catenin signaling pathway, promotion of apoptosis, and inhibition of breast cancer cell proliferation, migration and invasion.

In BC, NEAT1, linc00617, OR3A4 and LINP1 lncRNAs are up-regulated and are implicated, through the decrease in E-cadherin expression, in the invasion and formation of metastases (88–91).

FGF14-AS2, an antisense lncRNA transcribed from the opposite strand of the FGF14 (fibroblast growth factor 14) gene, suppresses osteoclast differentiation *in vitro* and osteolytic bone metastasis of BC *in vivo*. FGF14-AS2, by reducing eIF4E/eIF4G complex formation and eIF4E phosphorylation, suppresses the translation of RUNX2 thereby reducing the transcription of RANKL, the master regulator of osteoclast differentiation. Decreased FGF14-AS2, caused by YTHDF2-mediated degradation, is significantly correlated with poor prognosis in BC patients (92) (**Table 1**).

### 4.3 Lung cancer bone metastasis

lncRNA PXN-AS1-L has been found up-regulated in non-small cell lung cancer (NSCLC) cell lines compared to normal ones, and this up-regulation was also observed in NSCLC cells isolated from metastatic site. The same result have been determined in human biopsies in NSCLC tissues: PXN-AS1-L was elevated in cancerous respect to noncancerous lung tissues and further up-regulation was observed in bone metastatic tissue. Moreover, the expression of PXN-AS1-L positively associated with that of PXN in NSCLC tissues, supporting the positive regulation of PXN by PXN-AS1-L and the importance of PXN in the oncogenic roles of PXN-AS1-L in NSCLC (93).

While the role of lncRNA metastasis-associated lung adenocarcinoma transcript 1 (MALAT1) in lung cancer has been widely analysed, its clinical significance in bone metastasis from lung cancer has been only recently studied. Liu and colleagues demonstrated that MALAT1 is up-regulated in NSCLC tissues with bone metastasis and in lung cancer cell lines with high bone metastatic tropism. Moreover, MALAT1 is involved in cell proliferation, migration, invasion, and tumorigenesis, while it inhibits apoptosis in NSCLC cells. The authors suggest the important role of MALAT1 in the induction of bone metastasis in NSCLC patients (94).

It has been reported that lncRNA SOX2OT is present and abnormally accumulates in exosomes derived from the peripheral blood of NSCLC patients with bone metastasis. Furthermore, the patients with higher expression of lncRNA-SOX2OT experienced a significantly shorter overall survival. The authors demonstrated that tumor-derived exosomal lncRNA-SOX2OT promotes bone metastasis of lung cancer by targeting the RAC1 signaling pathway through miRNA-194-5p in osteoclasts and regulating the TGF-β/pTHrP/RANKL signaling pathway in osteoclasts. In summary, these results indicate that exosomal lncRNA-SOX2OT could potentially function as a powerful prognostic biomarker (95).

Exosomal lncRNA-HOTAIR derived from A549 and H1299 lung cancer cells promotes osteoclast differentiation and bone resorption by targeting TGF-β/PTHrP/RANKL pathway (96) (**Table 1**).

## 5 lncRNA as novel biomarkers in diagnosis and prognosis of cancer

The current diagnostic methods for cancer are expensive or invasive, such as MRI, ultrasound molecular pathology from tissue biopsies, detection of circulating tumour cells. Additionally, their prognostic potential, in term of prediction of cancer and metastasis onset, is limited. Therefore, to reduce cancer-related mortality it is important to identify early diagnostic biomarkers already measurable before the tumour onset. In the last decades, advances have been made in implementing ncRNAs, especially miRNAs, as new tools with high diagnostic value for screening (99).

Aberrant expression profiles of lncRNAs have been identified in several diseases, especially in cancer. They modulate either oncogene or tumour suppressive genes, thus affecting tumour development, and lncRNA signatures have been proposed to identify and classify different types of cancer. Thus, lncRNAs represent a new class of biomarkers with high potential in diagnosis and prognosis but also as novel, and possibly more effective, therapeutic targets.

Different approaches to therapeutically target lncRNAs are under investigation.

The classical CRISPRs (Clustered Regularly Interspaced Palindromic Repeats)/Cas9 system, a genome editing tool for molecular biology, can be used for the transcriptional inhibition of regions of interest in lncRNA loci. CRISPR is also being investigated to restore the expression of lncRNAs with tumor suppressor activity. CRISPR activation, CRISPRa, and CRISPR interference, CRISPRi, can be used to overexpress or silence lncRNA expression *via* catalytically dead Cas9 (dCas9).

More recent is the use of the CRISPR/Cas13 system to block lncRNAs in cancer. Cas13 can be located throughout the cell and, in this way, it has the potential to break down both nuclear and cytoplasmic lncRNAs.

Therefore, CRISPR/Cas system not only gives the possibility to better understand the functionality of lncRNAs but also provides innovative ways to investigate the potential therapeutic roles of lncRNAs through different approaches (100).

However, research efforts must be encouraged to depict better delivery strategies that might increase the success rate of delivery of CRISPR-Cas components in cancer cells.

Among other mechanisms for interfering with the lncRNA system, there are molecules used to post-transcriptionally knock down lncRNAs that are overexpressed in tumors: the oligonucleotide-based therapy with the single-stranded antisense oligonucleotides (ASOs) and the double-stranded RNA-mediated interference (RNAi) (17).

Since the expression of lncRNAs is generally tissue-specific, this feature needs to be exploited to therapeutically target the lncRNAs in systemic treatment for human diseases.

lncRNAs could also potentially function as biomarkers to predict the risk to develop metastasis in patients with cancer. lncRNAs can be used as indexes to control the progression from primary to metastatic disease and, consequently, can be regarded as both diagnostics tools and therapeutic targets of different types of cancers.

Importantly for their implementation as biomarkers, lncRNAs have been detected, other than in tissues, also in serum, plasma and other biological fluids (urine, saliva, synovial liquid), where lncRNAs are highly stable. Therefore, their assessment may be regarded as non-invasive, or minimally invasive (101).

### 5.1 lncRNAs as diagnostic biomarkers

Contrarily to miRNAs, lncRNAs possess a high degree of specificity for tissue type and disease, becoming ideal candidates for cancer diagnosis.

The best-known lncRNAs used for diagnosis of cancer is PCA3 (Prostate Cancer Antigen 3, also known as DD3) for the diagnosis of prostate cancer. This well-investigated lncRNAs is strongly upregulated in more than 90% of prostate tumours and metastasis compared to normal prostatic tissue (102, 103). Given its specificity for tumour tissue compared to healthy one and that its undetectability in other tissues, PCA3 is the most specific gene for prostate cancer known so far. It can be detected in urine and its specificity is even superior to that of prostate specific antigen (PSA, the gold standard biomarker for prostate cancer diagnosis) and of digital rectal examination (104). Urinary PCA3 detection is the first urinary-based molecular diagnostic test approved by Food and Drug Administration (FDA) and, nowadays, it is widely used for the diagnosis of prostate cancer (105). Importantly, urinary PCA3 levels do not correlate with prostate volume but correlate with tumour aggressiveness as described by the Gleason score, thus reflecting the aggressiveness of prostate cancer (106). Other lncRNAs have been studied or implemented for the diagnosis of prostate cancer. MALAT-1, which is overexpressed during prostate cancer progression, was detected in plasma of patients with prostate cancer compared to non-prostate cancer patients. With a sensitivity of 58.6% and specificity of 84.8%, this lncRNAs has been proposed as a biomarker for prostate cancer diagnosis. Its detection in plasma increases the diagnostic accuracy of prostate cancer for patients with abnormal levels of serum PSA, preventing unnecessary invasive procedure of diagnosis such as biopsies (107). lncRNA PCAT-18 has been detected in plasma and can be considered a potential biomarker for metastatic prostate cancer as its expression levels increase progressively as cancer progress from localized to metastatic (108). Urinary detection of lncRNA-p21 can be used to discriminate between prostate cancer and benign prostate hyperplasia, as its levels were significantly higher in prostate cancer patients. By analysing the ROC curve, lncRNA-p21 specificity increased significantly when combined with serum PSA (109).Other lncRNAs have shown some predictive potentials for prostate cancer diagnosis alone or in combination (110).

Breast cancer conventional serum biomarkers are carcinoembryonic antigen (CEA) and cancer antigen 15-3 (CA15-3), however their clinical use raised several concerns regarding their low sensitivity and specificity. The lncRNA RP11-445H22.4 was found highly expressed in both BC tissue and serum of BC patients compared to controls. It showed a sensitivity and specificity of 92% and 74% with an AUC of 0.904, better values than that of common breast tumour biomarkers and of traditional ultrasound diagnostic methods (111), suggesting lncRNA RP11-445H22.4 as a potential biomarker with enhanced diagnostic value for BC. Other lncRNAs, reviewed elsewhere (20, 112, 113), are under investigation as potential markers for diagnosis of BC, however the majority of them are of tissue origin, which makes them less available especially with non-invasive methods. Additional studies are needed to identify potential circulating biomarkers for BC diagnosis.

A screening of lncRNAs abnormally expressed in plasma of NSCLC patients led to the identification of three lncRNA SPRY4-IT1, ANRIL and NEAT1, all displaying high sensitivity and specificity for NSCLC. However, when combined their diagnostic value dramatically increased with higher sensitivity and specificity than the three alone. These results suggested that the circulating levels of SPRY4-IT1, ANRIL and NEAT1 can serve as a signature for the prediction of NSCLC (114). Also, MALAT1, detected in whole blood samples, was overexpressed in patients with NSCLC compared to healthy volunteers with an AUC of 0.79, indicating that circulating MALAT1 can represent a diagnostic tool for NSCLC with high specificity and low invasiveness (115).

Other lncRNAs are under study as potential biomarkers for diagnosis of other cancers, such as lncRNA LINC00152 for hepatocellular carcinoma, lncRNA H19 for gastric cancer, lncRNA UCA1 for bladder cancer and oral squamous cell carcinoma, MEG 3 for multiple myeloma, etc. (101, 110). It is important to stress that, although all the lncRNAs described here have a strong diagnostic potential, PCA3 is the only one recommended for human use.

### 5.2 lncRNAs as prognostic biomarkers

lncRNAs display also a prognostic potential for several cancers. A prognostic biomarker identifies the probability of a certain clinical event, disease outcome, or disease progression in patients already affected by a disease or a medical condition (116). Considering this definition, those lncRNAs up- or down-regulated in cancer patients that can correlate with tumor grade and stage might represent good candidates as prognostic markers. Indeed, some lncRNAs are associated with cancer cell proliferation, invasion, EMT, and metastasis, but other can be associated to metastatic dormancy (15, 117). In BC, lncRNAs can discriminate between different types of BC. For example, lncRNA NAS1 expression was found to be associated with hormone receptor expression and with late recurrence of ER+ BC, suggesting its potential as a biomarker for ER+ BC (118). In a study, HOTAIR expression correlated with lymph node metastasis from TNBC and with androgen expression receptor, proposing HOTAIR as a potential prognostic marker correlated with different therapeutic strategies for patients with a specific subtype of TNBC (119). MALAT1, one of the most studied and controversial lncRNA, overexpression in a mouse model inhibited BC metastasis. On the other hands, inactivated MALAT1 promoted lung metastasis, which was reverted with its restoration (120). An up-to-date summary of lncRNAs with prognostic value in BC has been recently published by Sobhani and colleagues (121). Notably, only few lncRNAs are prognostic for metastasis (122) occurrence and none for bone metastasis from BC.

Other lncRNAs have been suggested as prognostic markers for prostate cancer, e.g., SChLAP1 that is associated with metastasis occurrence (78), or lung carcinoma, e.g., HOTAIR for small cell lung cancer, which associate with lymphatic invasion and relapse (122). However, none of the lncRNAs under studies in cancer patients has been approved for routine prognosis.

### 5.3 Pre-analytical variables that affects circulating lncRNAs implementation as biomarkers

The unique characteristics of lncRNAs have opened the door to the possibility of their use as markers for cancer diagnosis and prognosis. However, much remains to be explored about their origins and functions before their implementation in diagnosis and treatment of cancer. Several challenges need to be solved to further develop circulating lncRNA-based diagnostics for clinical applications.

The first issue, similar to all biomarkers in general, concern those pre-analytical variables that can affect the reliability of the measurements, such as the choice of starting material (whole blood, plasma, serum, urine etc.), storage methods, donor-related and environmental factors (e.g. inflammation status, physical activity status, age, diet, confounding diseases, pharmacological treatments, etc.) (123). This issue has not been still investigated and the identification of the pre-analytical warnings to be considered in lncRNA assessment is of fundamental relevance for the establishment of their diagnostic usefulness.

Another important challenge, in the implementation of lncRNAs as biomarkers, concerns the methods to be used for RNA isolation and measurements. Different extraction methods have been used in studies of circulating lncRNAs, namely column-based methods and phenol/chloroform-based protocols, the latter having the principal drawback of possible organic and phenolic contaminants in the purified RNA extract. Also the methods for quantification of the extracted material may rise some concerns as the most commonly used instruments, such as the NanoDrop spectrophotometer, is not particularly sensitive in the case of plasma/serum-derived RNAs (101). Thus, comparison of different extraction methods, quantification system and standardization are still necessary.

Several molecular tools are used to detect and quantify circulating lncRNAs that span from qRT-PCR to RNA-seq, RNA-FISH or lncRNAs microarray platforms. While qRT-PCR analysis is featured by high costs per sample, it has a low throughput, and has the limit of detection of only annotated lncRNAs, RNA-seq have the advantage that permit the identification of both known and new lncRNAs and the cost per sample can be lowered thanks to sample multiplexing assay. However, expert personnel for the data analysis and specific equipment are necessary for lncRNA measurement with RNA-seq, making this technique, even though very promising, still not easily accessible for laboratory analysis. Finally, data normalization strategy still poses several challenges, as it is a critical step for reproducibility and comparison for results performed by different laboratory or with different platforms or from different samples (101).

Therefore, the implementation of standard operating procedure (SOPs) related to isolation, measurement and analysis of lncRNAs is mandatory for translating single or signatures of lncRNAs in clinically relevant tests.

Standardization of methodologies, normalization procedures and sample processing is important to reduce inter-laboratory and inter-user variability. With these attentions, it will be possible also to compare existing and different studies to univocally identify lncRNAs as novel specific, sensitive and reliable cancer biomarker.

## 6 Conclusions

Improvements in surgery and chemotherapy have provided new support for tumor growth control and, currently, metastases remain the primary concern. The researchers’ efforts are aimed at finding new mechanisms to counter the development of metastases, or to keep them in a dormant state. The challenges for the next decade are to find and develop new, long-lasting, affordable and effective therapies to improve the survival of cancer patients.

The discovery of ncRNAs not only adds new players to the complicated puzzle of cancer development, but also increases the available possibilities for countering its progression.

In particular, research is accelerating to decipher the exact molecular mechanisms involving lncRNAs in the development, growth and spread of cancer and in the prediction of clinical outcomes. In particular, the implication of lncRNAs in the formation and development of bone metastases has become an emerging and promising approach to consider for patients with this aggressive disease.

In the era of precision medicine, therefore, lncRNAs can be used as biomarkers and targets in cancer management. Although the investigations in this field are still at a beginning phase some interesting, and in some cases promising, results have been obtained so far. In the case of the most common tumors, e.g., breast, prostate, and lung cancers, single lncRNAs or lncRNA-signatures have been identified as associated with tumor onset and/or metastasis development. The high level of tissue specificity of lncRNA expression represent a great advantage and have allowed a univocal association with cancer types and/or cancer stages.

On the other side, being lncRNA involved in basic cellular processes, there is the possibility that a single species may be involved in the general pathophysiological mechanisms underlying tumorigenesis or metastasis development. This is the case of MALAT1 whose transcript is retained in the nucleus where it forms molecular scaffolds for RNP complexes; it may act as a transcriptional regulator for numerous genes, including some genes involved in cancer metastasis and cell migration, and it is involved in cell cycle regulation. Its upregulation in multiple cancerous tissues has been associated with the proliferation and metastasis of tumor cells and, therefore, it can be regarded as a pan-tumor marker.

Beside the undoubtable central role in cancer biology, the applicability of lncRNA-based assays in clinical setting is still very far since several technical issues should be solved, from the pre-analytical variables to be taken into account for their measurement, to the most reliable method and the post-analytical data analysis to be determined.

## Author contributions

Conceptualization and original draft preparation, PM. Writing, review and editing, PM, MG and GL. All authors have read and agreed to the published version of the manuscript.
